# Glutamate signaling through the NMDA receptor reduces the expression of scleraxis in plantaris tendon derived cells

**DOI:** 10.1186/s12891-017-1575-4

**Published:** 2017-05-25

**Authors:** Christoph Spang, Ludvig J. Backman, Sandrine Le Roux, Jialin Chen, Patrik Danielson

**Affiliations:** 1 Department of Integrative Medical Biology, Anatomy, Umeå University, SE-901 87 Umeå, Sweden; 2 Department of Clinical Sciences, Ophthalmology, Umeå University, Umeå, Sweden

**Keywords:** Glutamate, NMDAR1, Plantaris tendon, Tendinopathy, Scleraxis

## Abstract

**Background:**

A body of evidence demonstrating changes to the glutaminergic system in tendinopathy has recently emerged. This hypothesis was further tested by studying the effects of glutamate on the tenocyte phenotype, and the impact of loading and exposure to glucocorticoids on the glutamate signaling machinery.

**Methods:**

Plantaris tendon tissue and cultured plantaris tendon derived cells were immunohisto-/cytochemically stained for glutamate, N-Methyl-D-Aspartate receptor 1 (NMDAR1) and vesicular glutamate transporter 2 (VGluT2). Primary cells were exposed to glutamate or receptor agonist NMDA. Cell death/viability was measured via LDH/MTS assays, and Western blot for cleaved caspase 3 (c-caspase 3) and cleaved poly (ADP-ribose) polymerase (c-PARP). Scleraxis mRNA (*Scx*)/protein(SCX) were analyzed by qPCR and Western blot, respectively. A FlexCell system was used to apply cyclic strain. The effect of glucocorticoids was studies by adding dexamethasone (Dex). The mRNA of the glutamate synthesizing enzymes *Got1* and *Gls*, and NMDAR1 protein were measured. Levels of free glutamate were determined by a colorimetric assay.

**Results:**

Immunoreactions for glutamate, VGluT2, and NMDAR1 were found in tenocytes and peritendinous cells in tissue sections and in cultured cells. Cell death was induced by high concentrations of glutamate but not by NMDA. Scleraxis mRNA/protein was down-regulated in response to NMDA/glutamate stimulation. Cyclic strain increased, and Dex decreased, *Gls* and *Got1* mRNA expression. Free glutamate levels were lower after Dex exposure.

**Conclusions:**

In conclusion, NMDA receptor stimulation leads to a reduction of scleraxis expression that may be involved in a change of phenotype in tendon cells. Glutamate synthesis is increased in tendon cells in response to strain and decreased by glucocorticoid stimulation. This implies that locally produced glutamate could be involved in the tissue changes observed in tendinopathy.

## Background

Glutamate is known as a central amino acid—being essential for cell function and protein biosynthesis—and as the major excitatory neurotransmitter in the central nervous system of mammals [1]. Recently, many studies have expanded its properties by detecting glutamate receptors in various non-neuronal cells [2, 3]. Furthermore, it has been observed that glutamate signaling can be involved in pathological conditions in several peripheral situations, e.g., arthritis and diseased bone [1, 4, 5].

Glutamate has also been linked to persisting chronic tendon pain (tendinopathy). High levels of free glutamate have been detected in painful Achilles and patellar tendons and in Tennis elbow [6–8]. Injections of glutamate into tendon tissue have shown to result in increased tendon pain [9]. Furthermore, the vesicular glutamate transporter 2 (VGluT2), enabling the cells to release glutamate via synaptic-like vesicles, and various glutamate receptors, have been found to be expressed by tendon cells (tenocytes) in the Achilles and patellar tendons [10–12]. Studies have measured elevated levels of these elements in situations with chronic tendon degeneration [12, 13]. Based on these findings, it has been suggested that there may be an autocrine/paracrine glutamate signaling by tenocytes, which can contribute to the tissue features occurring in tendinopathy [10–12]. The ionotrophic N-Methyl-D-Aspartate (NMDA) receptor has been in focus in most of these studies. It is basically a heteromeric complex consisting of four subunits deriving from three different protein families (NMDAR1, NMDAR2 and NMDAR3) [14]. The composition of these subunits can vary in different cell types and affects the functional properties. The subunit 1 (NMDAR1) is known to be present in all possible NMDA receptor complexes in all cell types where NMDA receptors have been found [14]. In two in vitro studies on tendon cells it has been reported that glutamate exposure can lead to increased caspase activity and reduced cell viability partly mediated via the NMDA receptor [13, 15]. This observation is of interest as apoptosis is a feature that has frequently been observed in tendons affected by chronic tendinopathy [16, 17]. Thus, it might be that glutamate plays an important role in this process. However, how exactly apoptotic processes relate to the pathomechanisms of tendinopathy is not clarified yet.

Based on the findings above, it seems that glutamate may be actively involved in the process of tissue degeneration observed in tendinopathic tendons. Surprisingly, nothing is known about how excessive straining, a major risk factor for tendinopathy, influences the expression of glutamate signaling components, including the NMDA receptor, glutamate producing enzymes and levels of free glutamate in tenocytes. Furthermore, the immediate effect of glucocorticoids, e.g., dexamethasone (Dex), needs to be addressed in this context. Glucocorticoids are frequently used in patients with tendinopathies [18]. However, recent studies have questioned its use and found that it can actually harm the tissue [18]. Little is known about the relationship between glucocorticoids and the glutamate signaling machinery. In a study on rotator cuff tendinopathy, up-regulated levels of NMDAR1 in the tendon tissue have been found several weeks after treatment with local glucocorticoids [19].

Tissue changes in tendinopathic tendons also include formation of fat, cartilage, and bone tissue [20]. Studies have shown that under certain conditions tendon stem cells can differentiate into non-tenocyte-like cells, such as adipocytes and osteocytes [21]. Scleraxis (SCX) is a transcription factor for tenomodulin, a type II transmembrane glycoprotein, which is predominantly expressed in tendons and ligaments [22]. It has been found that scleraxis overexpression promotes the differentiation of mesenchymal stem cells into tendon progenitor cells [23]. Furthermore, reduction of scleraxis has been shown to inhibit the differentiation of rat tendon stem cells into tenocytes [24]. In addition, tenomoduline knock-out results in decreased self-renewal of tendon stem cells [25, 26]. It is, however, not known if signaling substances such as glutamate have an impact on these features, including a potential impact on scleraxis production and the phenotype of tendon derived cells.

In patients with midportion Achilles tendinopathy, the importance of the plantaris tendon has recently been highlighted. It has been shown that the plantaris tendon can be positioned very close to the Achilles tendon, the two tendons seemingly interfering with each other, in a subgroup of patients [27, 28]. These plantaris tendons often exhibit a degenerative tissue morphology, which indicates a co-existing plantaris tendinopathy [29, 30]. Nevertheless, there is nothing known about a possible occurrence of a glutamate signaling machinery in these plantaris tendons or in the peritendinous connective tissue in between the Achilles and the plantaris tendons.

The aim of the present study was to examine the expression patterns of glutamate, NMDAR1 and VGluT2 in the plantaris tendon and the adjacent peritendinous connective tissue in midportion Achilles tendinopathy patients. Furthermore, the possible effects of glutamate receptor stimulation (with NMDA or glutamate) on tendon derived cells in vitro, including changes in scleraxis expression, were evaluated. Finally, it was analyzed if the glutamate signaling machinery changes in response to excessive strain and to exposure of the corticosteroid Dex.

## Methods

### Patients and surgical treatment

All patients included in this study had suffered from chronic midportion Achilles tendinopathy with a duration of pain for at least 3 months. Tendon material from 17 patients (4 women, 13 men; mean age 50 years; range 21–69 years) was examined.

All patients underwent a previously described surgical procedure [31, 32], including the removal of the plantaris tendon and scraping of the ventro-medial aspect of the Achilles tendon. The excised plantaris tendons (*n* = 17) with attached peritendinous connective tissue were then further processed. Most tendons (*n* = 15) were used for tissue analysis using immunohistochemistry. From four of these fifteen tendons, parts were also processed for cell culture. Material from four patients was also used for in situ hybridization. From two patients, tendon material was only used for culturing purposes (see below).

### Tissue processing for immunohistochemistry

Immediately after surgery, the tissue samples were fixed in 4% formaldehyde in 0.1 M phosphate buffer (pH 7.0) at 4 °C. On the next day the tendon samples were washed three times in Tyrode’s solution containing 10% sucrose at 4 °C. The first wash was performed overnight. Eventually the specimens were mounted on a thin cardboard in optimal cutting temperature (OCT) embedding medium (code: 45830; Miles Laboratories, Naperville, IL, USA) and frozen at −80 °C until sectioning. The frozen tissue specimens were then cut using a cryostat with a thickness of 7 μm and mounted on super frost slides (code: 041200; Menzel Gläser, Braunschweig, Germany).

Sections from plantaris tendons and adjacent peritendinous tissue were immunohistochemcially stained for glutamate, NMDAR1, phosphorylated NMDAR1 (pNMDAR1) and VGluT2. The staining procedure followed an established protocol, which has previously been described in several articles [30, 33]. The incubation of primary antibodies was performed for 60 min at 37 °C.

Glutamate was detected by using a polyclonal rabbit antibody (code: G-143; RBI, Natrick, MA, USA) at a dilution of 1:20. For detecting NMDA receptors, two antibodies against subunit 1 (NMDAR1), which is known to be present in all possible NMDA receptor complexes [14], were used separately. One was a rabbit polyclonal antibody from Abcam (1:50; code: ab134308; Cambridge, UK) and the other was a goat polyclonal antibody from Santa Cruz (1:50; code: sc-1467; Santa Cruz, CA, USA). The antibody for visualizing pNMDAR1 was a goat polyclonal from Santa Cruz (1:50; code: sc-12890). VGluT2 was detected with two goat polyclonal antibodies. One was from Abcam (1:100; code: ab101760) and the other from Santa Cruz (1:50; code: sc-26026). For visualizing scleraxis, a rabbit polyclonal antibody from Abcam was used (1:25; ab58655).

For the rabbit antibodies, normal swine serum (code: 014-000-121; Jackson I.R., West Grove, PA, USA), for blocking, and swine anti rabbit secondary antibody conjugated with tetramethylrhodamine isocyanate (TRITC) (code: R0156; DAKO, Glostrup, Denmark) were used. For goat antibodies, donkey normal serum (code: 017-000-121; Jackson I.R.), for blocking, and donkey anti goat secondary antibody conjugated with fluorescein isothiocyanate (FITC) (code: 705-095-147; Jackson I.R.) were used.

For control purposes, stainings replacing the primary antibody with PBS were performed. The specificity of the primary antibodies had been tested on human tendons in previous articles of our group including preabsorption experiments [10, 30, 33].

The slides were analyzed using a Zeiss Axioskop 2 plus microscope equipped with epifluorescence and an Olympus DP70 digital camera.

### In situ hybridization

In situ hybridization for detecting VGluT2 mRNA was accomplished according to an established protocol [34]. On a subset of specimens (n = 4) a digoxigenin (DIG)-hyperlabeled oligo-nucleotide probe (ssDNA) was used. The sequence of the antisense probe was CCTTG TACAA ATTCC TCTTT CTTTT CCCAA CCACT AGGCC AACCT CCA (GeneDetect, Auckland, New Zealand). This sequence is complementary to nucleotides 2066 to 2113 of the coding sequence of human VGluT2. An alkaline phosphatase-labeled anti-DIG antibody (code: 11093274910; Roche, Germany) was then applied to detect the mRNA. As a positive control probe, an antisense probe recognizing β-actin in all species (code: GD5000-OP) was used (GeneDetect, New Zealand). The corresponding sense DIG-hyperlabeled ssDNA probe was used in previous studies [10, 33]. The reliability concerning VGluT2 mRNA for human tendon tissue by using the current ssDNA probe was hereby verified [10].

### Tissue processing for cell culture

Plantaris tendons processed for cell culture were kept in Dulbecco’s Modified Eagle Medium (D-MEM; code: 11960; Invitrogen) supplemented with 10% Fetal Bovine Serum (FBS; code: 25030; Invitrogen), 1% penicillin-streptomycin (pen strep) (code: 15410; Gibco) and 0.2% L-Glutamine (code: 25030; Invitrogen) when transported to the laboratory. The tendon samples were then thoroughly washed under sterile conditions in Hank’s Balanced Salt Solution (HBSS) (code: 14170; Invitrogen). Any parts of peritendinous tissues were carefully released. After that, the samples were cut into small pieces and enzymatically digested overnight at 37 °C using collagenase (Clostridiopeptidase A; code: C-0130; Sigma) in D-MEM in a concentration of 2 mg/ml. The products were then centrifuged and the supernatant was discarded. The pellet was dissolved in D-MEM with 10% FBS, 1% pen strep and 0.2% L-Glutamine and then cultured in a petri dish being placed in a humidified atmosphere of 5% CO_2_ in air and 37 °C. Medium was replaced every 48 h. Confluent cells were scraped and transferred into a culture flask. When these cells reached confluence, they were enzymatically detached using 0.05% trypsin (code: 15400; Invitrogen) in HBSS and split into 1:3 ratio. Cells were kept in liquid nitrogen until needed. All cells used in this study were from passage 2–4.

### Immunocytochemistry

15,000 cells/well were seeded on 8-well chamber slides (code: 354118; BD Falcon) overnight before being stained for Glutamate, NMDAR1, pNMDAR1, VGluT2, and scleraxis. Cells were initially fixed in 2% paraformaldehyde in 0.1 M phosphate buffer (pH 7.4) for 5 min and then washed 4 times for 1 min in 0.01 M phosphate buffered saline (PBS, pH 7.4). Then cells were blocked with normal serum (1:20) for 15 min and incubated with the primary antibody for 60 min at 37 °C. After that cells were washed again, blocked, and incubated with the secondary antibody for 30 min at 37 °C. Finally, cells were washed again and mounted using mounting medium including DAPI (code: P36962; Life Technologies).

Primary antibodies, normal serum and secondary antibodies were the same for immunocytochemistry as for immunohistochemistry. Control stainings using PBS were performed in the same way as for immunohistochemistry.

### Stimulation with glutamate/NMDA

One purpose of this study was to examine the effect of glutamate related activation of the NMDA receptor in tendon derived cells. The cells were seeded in 6 well plates (code: 83.3920; Sarstedt, Germany) overnight and starved in in D-MEM supplemented with 1% FBS for 24 h. Stimulation with glutamate (code: G8415-100G; Sigma) or NMDA (code: M3262-100MG; Sigma) was performed. NMDA is a prototypic agonist that binds specifically to the NMDA receptor but not to other glutamate receptors [35], and has previously been used in cell culture studies [36]. During the stimulation period the cells were kept in D-MEM with or without supplementations (FBS, pen strep, L-Glutamine). Medium containing the stimulating substances were changed every day. The acid nature of glutamate and of HCl, in which glutamate was dissolved, was compensated by titrating NaOH to reach a pH between 7.30 and 7.35 in the medium solutions that were applied to the cells. The same was done for all solutions containing NMDA. Stimulation was performed for up to 3 days. Glutamate and NMDA were added to get final concentrations ranging from 1 μM to 10 mM when evaluating the impact on cell viability via LDH and MTS Assays (see below). For the analysis of proteins and mRNA concentrations were used up to 500 μM for NMDA and up to 1 000 μM for glutamate. LDH and MTS Assays were performed after 24, 48 and 72 h of stimulation. The expression of cleaved caspase 3 (c-caspase 3) and cleaved PARP (c-PARP) using Western blot was determined after 24 h of NMDA stimulation. After 8 h cells were processed for qPCR to determine mRNA expression level for scleraxis (*Scx*) (see below). Scleraxis protein (SCX) was measured 24 h after stimulation via Western blot.

### Mechanical loading of tendon cells using a FlexCell system

One day before the experiments, the cells were seeded on a Bioflex culture plate membrane, which was pre-treated with collagen I (BF-3001C; Bioflex). The density was chosen to be 250,000 cells per well. Strains were then applied equibiaxially to the cells by a vacuum-induced deformation of the membrane downwards around a loading post. The daily loading procedure lasted for 120 min and included 10% strain with a frequency of 1 Hz. Cells were kept in 1% FBS in D-MEM during the whole procedure. Medium was changed before the loading. The procedure was repeated for 3 days, one set of loading every day. For more practical details, see [37]. Two hours after the last loading procedure on day 3 cells were further processed for qPCR in order to measure RNA levels of glutamate producing enzymes (Glutaminase, *Gls*; glutamic-oxaloacetic transaminase 1, *Got1*). Furthermore the amount of NMDAR1 protein was analyzed via Western Blot and the levels of glutamate were detected via a glutamate colorimetric assay.

### Stimulation with dexamethasone (Dex)

To evaluate the impact of glucocorticoids on the glutamate signaling components, the dexamethasone (Dex) (Sigma; code: D4902) was diluted in pure ethanol and then applied in concentrations ranging from 1 to 1000 nM. As a control untreated wells were stimulated with pure ethanol (vehicle control). The durations of stimulation varied between 6 and 48 h. Cells were then analyzed for *Gls* and *Got1* mRNA, for levels of free glutamate and NMDAR1 protein.

### Measuring cell death (LDH assay)

In order to measure cell death as a response to the stimulation with glutamate and NMDA, we used a lactate dehydrogenase assay from Promega (code: G1780). At each time point supernatant was collected and stored in −80 °C until all time-points were collected. For the analysis 50 μl of the sample was pipetted into a 96-well plate and mixed with 50 μl reconstituted substrate mix. Then incubation for 30 min in a light protected condition followed before 50 μl of stop solution was added. Finally the absorbance was read at 490 nm.

### Measuring cell viability (MTS assay)

The effect of NMDA and glutamate on cell viability was measured using a MTS assay (CellTiter 96® Aqueous One Solution Cell Proliferation Assay; code: G3581; Promega). Cells were seeded in a 96 well plate overnight at a density of 5000/well. For the analysis MTS reagent (20 μl per 100 μl media) was added and then incubated for 4 h at 37 °C, 5% CO_2_. The amount of formazan produced by cellular reduction of MTS, was analyzed by a micro-plate reader at the absorbance of 490 nm.

### Glutamate assay

After 24 h of Dex exposure or 3 days after strain, cells were lysed in RIPA lysis buffer. Levels of free glutamate were measured using a colorimetric glutamate assay kit from Abcam (code: 83389) according to the manufacturer’s specifications. The Assay was normalized to the total amount of proteins using total proteins using Protein Assay Dye Reagent Concentrate (code: 500-0006; Bio-Rad) with Bovine Albumin Serum (BSA; code: A9647; Sigma) as a standard.

### Western blot

Cells were washed in sterile PBS and then scraped in lysis buffer (RIPA) supplemented with a protease and phosphatase inhibitor cocktail (100X, code: 78440; Thermo Fisher Scientific) (1:200), then put in an Eppendorf tube and incubated on ice for 30 min. After that the tube was centrifuged to remove cell debris. The supernatant was collected and analyzed for concentrations of total proteins using Protein Assay Dye Reagent Concentrate as a standard.

Before loading onto a SDS-PAGE gel, samples were, in the same concentrations, boiled in 2× Lammeli buffer (code: 161-0737; Bio-Rad) supplemented with beta-mercaptoethanol. After the electrophoresis (160 V, 60 min) the proteins were transferred to a polyvinylidene fluoride transfer membrane (PVDF code: sc-3723; Santa Cruz) for 1 h at 100 V. The membrane was then blocked with either 5% BSA or 5% non-fat milk powder in TBS-T for 60 min and finally incubated with the primary antibody overnight at 4 °C. On the next day the membranes were washed in TBS-T (3x5 min) and after that incubated with the secondary antibody at room temperature for 60 min. After the final wash the membranes were treated with chemiluminescent HRP substrate (code: RPN2232; GE Healthcare) for 5 min and then visualized using Odyssey® Fc imaging system (LI-COR, Lincoln, NE, USA). Quantification of pixel intensities (densitometry) was accomplished using Image J analysis software (NIH) (see Figs. 7c and 8d). Intensity of the protein of interest was divided by the intensity of β-actin for each group and then compared.

Antibodies were used against NMDAR1 (mouse; code: ab134308, abcam, UK), scleraxis (rabbit; code: ab58655; Abcam), c-PARP (code: 9541, Cell Signaling Technology), and c-caspase 3 (code: 9662P; Cell Signaling Technology), all in a dilution 1:1000 in 5% blocking medium. Beta-actin (code: 4967; Cell Signal) was used to confirm equal loading. The secondary antibodies used were from Cell Signal (codes: 7074S, 7076S).

### RNA isolation, reverse transcription, qPCR

For the extraction of RNA, an extraction kit was used (code: 74106; Qiagen) and the protocol from the manufactures was followed.

The isolated RNA was then reverse transcribed into cDNA via a High Capacity cDNA Reverse Transcription kit (code: 4368813; Applied Biosystems). The settings for the conversion were 10 min at 25 °C followed by 120 min at 37 °C, 5 min at 85 °C and then being paused at 4 °C on a thermal cycler (Eppendorf Mastercycler EP Gradient S).

The quantitative PCR (qPCR) was accomplished using TaqMan fast universal PCR mastermix (code: 4352042) and probes for *Scx* (code: Hs03054634; Applied Biosystems), *Gls* (code: Hs00248163; Applied Biosystems), and *Got1* (code: Hs00157798; Applied Biosystems). 20 ng of cDNA was used. The amplification was performed in a ViiA7 Real-Time PCR system (Applied Biosystems). The expression levels of genes was calculated in relation to that of *beta actin* (code: 4352935; Life Technology). For more information on practical details see [37].

### Statistics

Data were analyzed with SPSS Statistics software (20.0; IBM, Chicago, IL, USA). One-way ANOVA with Bonferroni post-hoc test (when comparing three groups or more) and independent samples *t*-test (when comparing two groups) were applied. Statistical significance was predetermined at p < 0.05. All experiments were successfully performed at least three times with cells samples from at least two different patients. All experiments were performed in groups of at least triplicate samples (i.e., at least 3 wells/group) and for analysis (e.g., qPCR) they were pipetted in duplicates.

## Results

### Expression patterns of the glutamate system

**Fig. 1:**
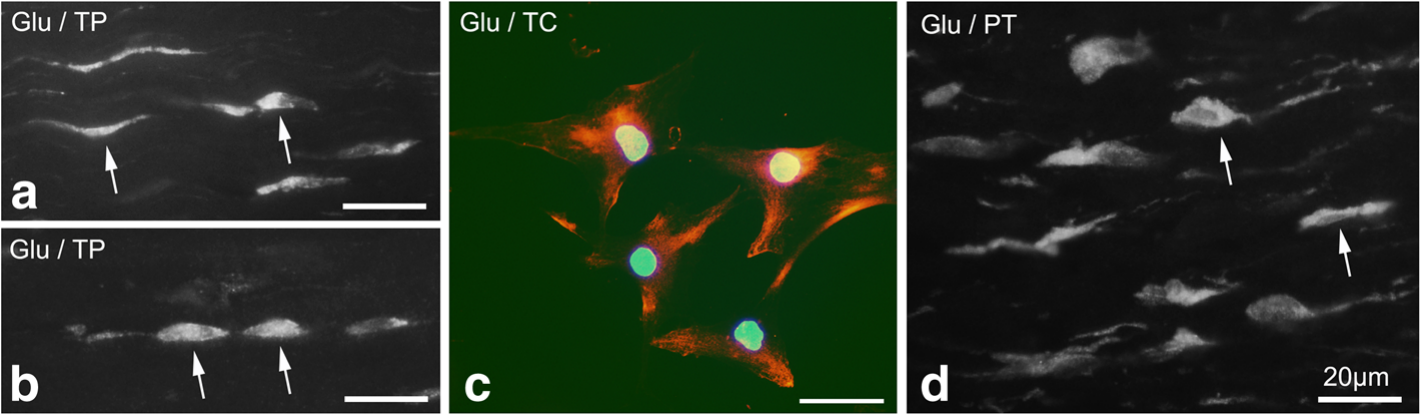
Plantaris tendon tissue proper (*TP*), plantaris peritendinous connective tissue (*PT*), and cultured tendon cells (*TC*) stained for glutamate (*Glu*): Examples of immunoreactions (*arrows*) are seen in slender and rounded tenocytes in the tendon tissue proper **a**, **b**, in cultured tendon cells (*red*; **c**) and in cells in the peritendinous tissue **d**. Nuclei in **c** are stained with DAPI (*blue*). *Bars* indicate 20 μm length

Immunoreactions for glutamate were seen in tenocytes in the tendon tissue proper (Fig. 1a, b), the cultured tendon derived cells (Fig. 1c), and in the cells in the peritendinous connective tissue (Fig. 1d).

**Fig. 2:**
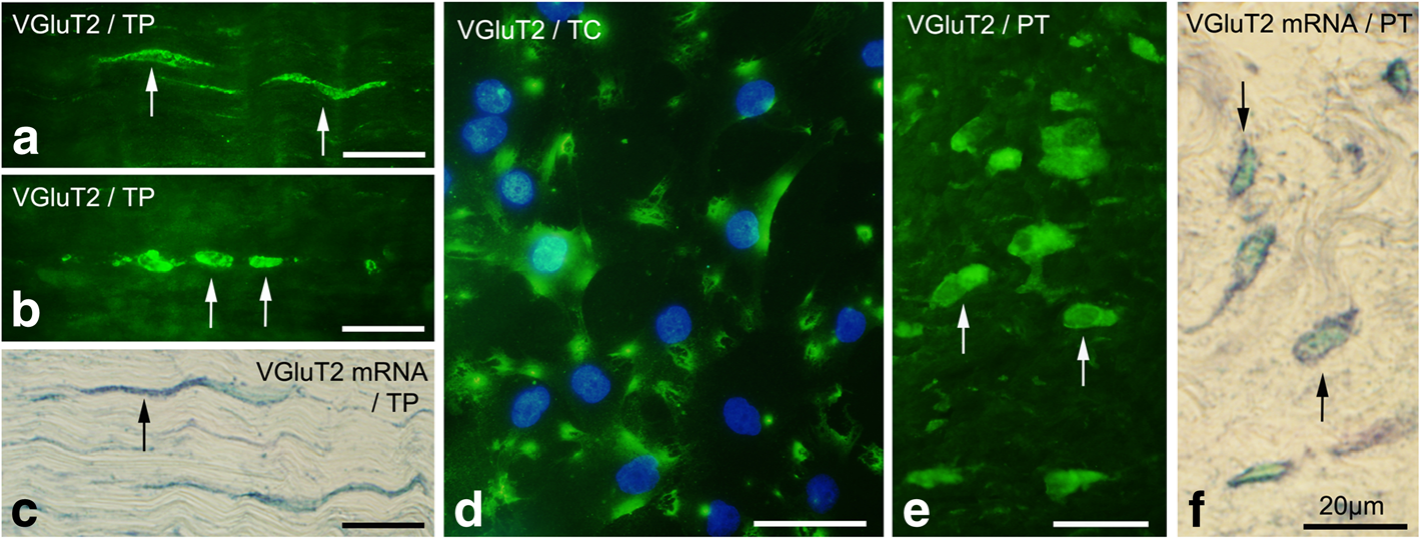
Plantaris tendon tissue proper (*TP*), peritendinous connective tissue (*PT*), and cultured tendon cells (*TC*) stained for VGluT2: Examples of immunoreactions (*arrows*) are seen in slender and rounded tenocytes in the tendon tissue proper **a**, **b**, in cultured tendon cells (*green*; **d**) and in cells in the peritendinous tissue **e**. VGluT2 mRNA was located in tenocytes **c** and peritendinous cells **f** in tissue sections, as verified by in situ hybridization. Nuclei in **d** are stained with DAPI (*blue*). *Bars* indicate 20 μm length

Vesicular glutamate transporter VGluT2 was also found to be expressed by tenocytes of the tendon tissue proper (Fig. 2a, b) and by cells in the peritendinous connective tissue (Fig. 2e) by immunohistochemistry. In situ hybridization revealed VGluT2 mRNA in tenocytes of the tendon tissue proper and in peritendinous cells (Fig. 2c, f, respectively). Tendon derived cells in vitro also expressed VGluT2; the immunoreactions being mostly located in the periphery of the cells (Fig. 2d).

**Fig. 3:**
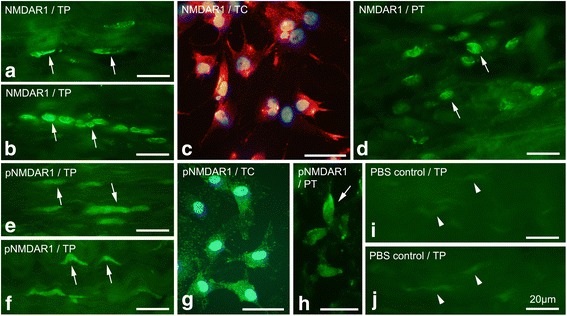
Plantaris tendon tissue proper (*TP*), peritendinous connective tissue (*PT*), and cultured tendon cells (*TC*) stained for NMDAR1 **a**, **b**, **c**, **d** and pNMDAR1 **e**, **f**, **g**, **h**: Examples of immunoreactions (*arrows*) can be observed in slender and rounded tenocytes in the tendon tissue proper **a**, **b**, **e**, **f**, in cultured tendon cells **c**, **g**and in cells in the peritendinous tissue **d**, **h**. Nuclei in **c** and **g** are stained with DAPI (*blue*). In **i** and **j**, control stainings replacing the primary rabbit **i** and goat **j** antibodies with PBS are shown. No positive reactions can be seen. *Bars* indicate 20 μm length

NMDAR1 immunoreactions were also detected in tenocytes of the tendon tissue proper (Fig. 3a, b), in cultured tendon derived cells (Fig. 3c), and in peritendinous cells (Fig. 3d). Like for NMDAR1, immunoreactions for the phosphorylated version of the receptor, pNMDAR1, were detected in tenocytes (Fig. 3e, f), cultured cells (Fig. 3g) and peritendinous cells (Fig. 3h). NMDAR1 protein was furthermore detected in cultured tendon cells using Western blot (see below).

VGluT2, NMDAR1, and glutamate immunoreactions in tendon tissue were especially seen in the cells that had an abnormal appearance such as rounded and wavy shaped.

Control stainings with PBS showed no reactions, neither when using swine anti-rabbit (Fig. 3i) nor donkey anti-goat (Fig. 3j) secondary antibodies.

### Cell viability and cell death

**Fig. 4:**
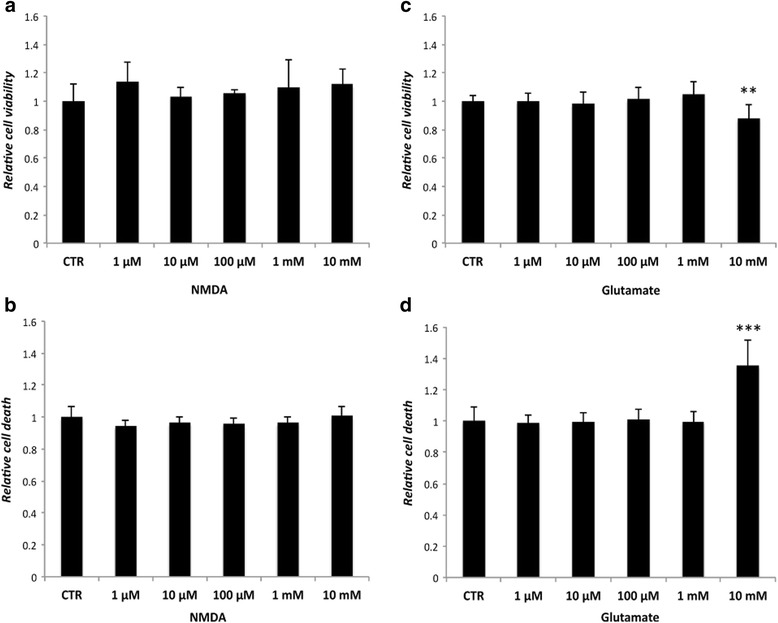
Cells treated with NMDA **a**, **b** and glutamate **c**, **d** for 72 h analyzed for cell viability (MTS Assay; **a**, **c**; *n* = 9) and cell death (LDH assay; **b**, **d**; *n* = 6). No difference occurs in the NMDA treated cells. Cells exposed to glutamate show decreased cell viability and increased LDH levels at a concentration of 10 mM compared to the control (*CTR*). *Error bars* show standard deviation. (** *p* < 0.01; *** *p* < 0.001)

There were no significant differences found in terms of LDH levels and cell viability between unexposed cells and cells stimulated with NMDA at any time point (Fig. 4a, b). For stimulation with glutamate, only a concentration of 10 mM lead to a reduction in cell viability and an increase of LDH after 72 h (Fig. 4c, d).

Western blot for c-PARP and c-caspase 3 showed an absence of these proteins 24 h after stimulation with NMDA (data not shown).

### Scleraxis expression and the effect of NMDA stimulation

**Fig. 5:**
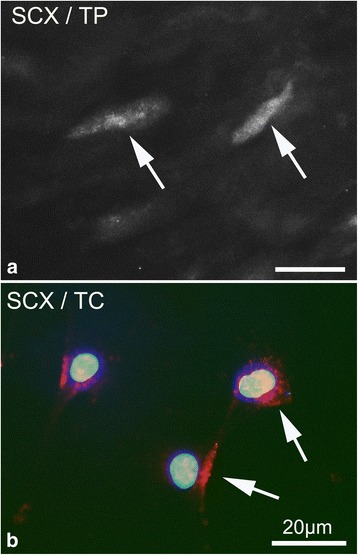
Plantaris tendon tissue proper (*TP*) in **a** and cultured plantaris tendon cells (*TC*) in **b** stained for scleraxis (SCX). *Arrows* indicate positive cells (*red* in **b**). Nuclei in **b** are stained with DAPI (*blue*). *Bars* indicate 20 μm length

Positive immunostainings for scleraxis were found in tenocytes in the plantaris tendon tissue proper (Fig. 5a) and in fibroblastic-like cells in the peritendinous tissue (not shown). Scleraxis was also found to be expressed by the cultured tendon derived cells from the plantaris tendon tissue proper (Fig. 5b).

**Fig. 6:**
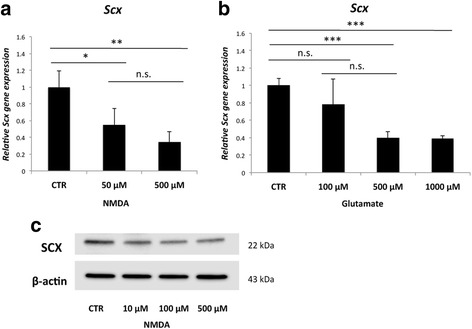
Results of NMDA **a** and glutamate **b** stimulation of cultured plantaris tendon derived cells on scleraxis gene (*Scx*) expression after 8 h. Scleraxis protein (SCX) after 24 h of NMDA stimulation is shown in **c**. A significant decrease in scleraxis mRNA can be seen after NMDA and glutamate stimulation. *Western blot* shows a seemingly dose-dependent decrease in scleraxis protein in cells treated with NMDA as compared to control (*CTR*). *Error bars* show standard deviation. (* *p* < 0.05; ** *p* < 0.01; *** *p* < 0.001; n.s. = not significant). *n* = 3

Scleraxis mRNA (*Scx*) was significantly decreased in cells treated with NMDA at concentrations of 50 μM and 500 μM (Fig. 6a) and with glutamate at concentrations of 500 μM and 1000 μM after 8 h as compared to unstimulated cells (Fig. 6b). There was less scleraxis gene expression in cells stimulated with 500 μM of NMDA as compared to cells stimulated with 50 μM. However, this difference was not statistically significant. The protein levels of scleraxis (SCX) were decreased after 24 h of NMDA stimulation, as shown by Western blot (Fig. 6c).

### Glutamate production and NMDAR1 protein after loading

**Fig. 7:**
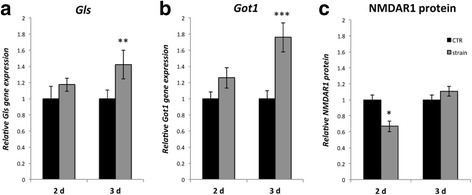
Results of 2 and 3 days of strain on the gene expression of *Gls* **a** and *Got1* **b**, as seen by *qPCR*, and the protein content of NMDAR1 as seen by *densitometry of western blot* **c**. After 2 days, there was a slight increase of *Gls* and *Got1* mRNA **a**, **b**. After 3 days this increase is more pronounced **a**, **b**. NMDAR1 is reduced after 2 days and slightly, but not significantly, increased after 3 days **c**. *Asterisks mark* significance for outcome in relation to the control (*CTR*). *Error bars* show standard deviation. (* *p* < 0.05; ** *p* < 0.01; *** *p* < 0.001). *n* = 3

The mRNA for the glutamate synthesizing enzymes *Gls* and *Got1* was significantly increased after 3 days of loading (Fig. 7a, b). Densitometry of western blots showed that the amount of NMDAR1 protein was decreased after 2 days but showed a tendency of increase after 3 days (not significant) (Fig. 7c). The levels of free glutamate did not show significant differences between loaded and unloaded cells (data not shown).

### Glutamate production and NMDAR1 protein after stimulation with dexamethasone

**Fig. 8:**
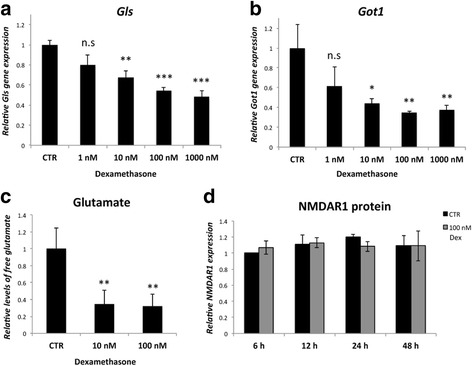
Results of 24 h of Dexamethasone exposure on gene expression (*qPCR*) of *Gls* **a** and *Got1* **b**, levels of free glutamate (*glutamate assay*) **c** and the protein content (*densitometry of western blots*) of NMDAR1 **d** in response to dexamethasone (*Dex*) exposure at concentrations from 1 nM to 1000 nM. *Gls* and *Got1* mRNA is reduced in a dose dependent manner **a**, **b**. Glutamate levels are decreased at 10 nM and 100 nM of dexamethasone. No changes can be observed for NMDAR1 **c**. *Asterisks mark* significance for outcome in relation to the control (*CTR*). *Error bars* show standard deviation. (* *p* < 0.05; ** *p* < 0.01; *** *p* < 0.001; n.s. = not significant). *n* = 3

The gene expression of *Gls* and *Got1* analyzed via qPCR showed a marked decrease after stimulation with Dex, which was found to be dose dependent (Fig. 8a, b). The levels of free glutamate were decreased at concentrations of 10 and 100 nM Dex (Fig. 8c). The NMDAR1 protein amount being measured via densitometry of the western blots was not affected by Dex within the examined time frame of 48 h (Fig. 8d).

## Discussion

The results of this study indicate that there is a glutamate signaling system in the plantaris tendon and the peritendinous connective tissue in between the plantaris and Achilles tendons in patients with midportion Achilles tendinopathy, as evident by the expression of glutamate, VGluT2, NMDAR1 and pNMDAR1. Cultured primary tendon cells harvested from plantaris tendons express the same components in vitro. We could detect cell death and a decrease of cell viability in the cultured tendon cells after 3 days of stimulation with glutamate. However, that observation was only made at a high concentration (10 mM). On the other hand, we found a decrease in scleraxis both on protein and RNA level after stimulation with NMDA and on RNA level after glutamate exposure. Loading increased the expression of the glutamate synthesizing enzymes *Gls* and *Got1*, whereas Dex decreased them. The levels of free glutamate were decreased after Dex exposure but did not alter after excessive strain. These findings represent new information and lead to new speculations concerning the role of glutamate in tendinopathy.

Chronic tendon disorders such as tendinopathies with degenerative tissue patterns are not fully understood. It has been speculated that locally produced signaling substances might be involved [38]. One of the discussed substances is glutamate. Expressions of VGluT2 and NMDAR1 have previously been observed in Achilles and patellar tendons, and were hereby found to be up-regulated in tenocytes exhibiting a rather pathologic appearance, i.e. being rounded or wavy in shape [10, 11]. The results of the present study show that these proteins are also present in the plantaris tendon, a tendon that has recently been highlighted to be involved in mid-portion Achilles tendinopathy [28]. Morphological studies have shown that there are often degenerative tissue features in the plantaris tendon when positioned very close to the Achilles tendon in situations of midportion Achilles tendinopathy [29, 30]. The results of the present study show that there is a glutamate signaling machinery with a possible autocrine/paracrine loop that might have an impact on the tissue changes that occur in tendinopathic tendons. Interestingly, in accordance with findings in the patellar and Achilles tendons [10, 11], we have found that reactions for the glutamate signaling components are especially expressed in cells that had an ‘abnormal’ rounded appearance, often seen in tendinopathic tendons and potentially showing rates of high metabolism. That might indicate that glutamate is highly involved in processes related to tendinopathic tissue changes. However, in this study the cells with rounded appearance have not been characterized and one cannot completely rule out the possibility that these cells are partly infiltrated inflammatory cell, synoviocytes or endothelial cells (see further below).

Previous in vitro studies on glutamate in relation to tendons have proposed an apoptotic effect of glutamate mediated via the NMDAR1 receptor [13, 15]. In our study, we could not detect any increased cell death after exposure with NMDA, a selective agonist of the NMDA receptor. Furthermore, there was no cleaved caspase 3 and no cleaved PARP present in the stimulated cells. Only at a concentration of 10 mM glutamate, there was a decrease in cell viability and an increase in LDH levels. In previous studies, cell death has been reported at glutamate concentrations of 500 μM [13], and 1.875 mM [15]. However, it should be recalled that in these referred studies, cells from different tendons and slightly different culturing conditions have been used. Therefore, the results of these studies are not inconsistent with the present findings. It is difficult to evaluate what concentrations in vitro reflect the concentrations found in vivo. In several microdialysis studies by Alfredson and co-workers [6–8] it has been found that in painful tendons the mean concentrations of glutamate was 196 μM for the Achilles tendon and 215 μM for the extensor carpi radialis brevis origin. Control tendons showed a significantly lower concentration (48 μM and 69 μM, respectively). Similar results were found for the patellar tendon. The concentrations detected appear to be much lower than those that were found to induce apoptosis in vitro. Thus, it can be questioned if glutamate concentrations in vivo indeed contribute to apoptotic processes in tendinopathy via the NMDA receptor in the tendon. In fact, the role of apoptosis in the pathomechanism of tendinopathy has not been elucidated yet. Further studies are needed to explore these pathways.

More importantly, our study shows that there is a down-regulation of scleraxis in cultured tendon derived cells after exposure to NMDA and glutamate. Scleraxis is a transcription factor that regulates the expression of tenomodulin, a transmembrane protein in tenocytes [22]. It is known that scleraxis is essential for the differentiation of mesenchymal stem cells towards tendon progenitor cells and tenocytes [23, 24]. Under certain conditions, tendon stem cells can become adipocytes and osteocytes [20, 39]. Tenocytes were found to have only very little of this differentiation capacity [39]. One can speculate that the decrease of scleraxis may lead, to some extent, to a reduced tenocyte phenotype. However, scleraxis is also expressed by other cell types and there are other markers that are highly expressed in tendon cells such as tenomodulin. In fact, not all mechanisms related to tenocyte differentiation are yet known. Nevertheless, it is reasonable to suggest that the NMDA/glutamate induced decrease of scleraxis seen in our study may be a mechanism that influences the process of tendinopathy. These aspects need to be examined in future studies.

Interestingly, in a previous study by our group we have observed the similar effect of scleraxis reduction in response to Dex stimulation [40]. Glucocorticoids are known to have harming affects on the tendon tissue, including higher risks for tendon ruptures [41], decreased mechanical properties [42], and decreased cell viability and collagen I production in vitro [43]. Our previous finding that Dex leads to a reduction of scleraxis, potentially making the cells less tenocyte like, might also contribute to the weakening of the tendon tissue. Some authors have suggested that there is a protein kinase dependent signaling pathway from the surface corticosteroid receptors to NMDA receptors [44, 45]. Thus it might be that the effect of Dex induced scleraxis reduction is mediated via the NMDA receptor. This very important and interesting aspect needs to be addressed in future research. Recent histological studies have found that glucocorticoids can lead to an increased expression of NMDAR1 in tendinopathy after several weeks [19]. In our current study, investigating a time frame of 48 h in vitro, we could show that this phenomenon is not an immediate effect. However, it could be speculated that based on the described possible structural connections between the receptors, there may be an up-regulation after long-term exposure of Dex. That might explain why studies on the clinical effect of glucocorticoids have reported short-term pain relief but no long-term benefits [46].

However, Dex may have different effects on cells infiltrating tendon tissues such as macrophages and synoviocytes. Several of these cell types are known to express NMDA receptors [47]. Therefore, future studies need to investigate the effect of Dex and the activation of glutamate receptors on other cell types present in tendon tissue. It is likely that different cell types show different responses. The role of glutamate signaling and its effect on tissue and cell changes related to tendinopathy may thus be more complex and may include other cells than resident tenocytes.

We furthermore show that loading of tendon derived cells results in an increase in glutamate synthesizing enzymes. This might further indicate that glutamate signaling is involved in chronic tendon overuse, which would be in accordance with the microdialysis results from Alfredson et al. [6–8]. However, it has to be recalled that the levels of free glutamate did not change after loading. This could be explained with the fact that glutamate is needed for protein synthesis and may therefore be metabolized very quickly. On the other hand it might be possible that glutamate is released as a signaling substance. The expression of NMDAR1 was found to be reduced after 2 days of loading, but increased after 3 days. That may indicate that continuous loading of tendon cells leads to a long-term up-regulation.

A drawback of this study is that the population of tendon derived cells used may not completely be homogenous. One should keep in mind that in vivo tendon tissue consists of other cell types than tenocytes, such as chondrocytes, endothelial cells, adipocytes, monocytes/macrophages and nerve cells [47, 48]. Even though it is likely that the rounded and wavy cells expressing high levels of glutamate and its receptor NMDA and transporter VGluT2 are transformed resident cells, no one has clearly proven this fact, as reliable markers for identification are lacking. Theoretically, it might be that they are infiltrating cells from the synovium or blood stream. Furthermore, it is also known that tendons consist of stem cells that harbor the potential to differentiate into osteocytes, chondrocytes and adipocytes [21, 39, 48].

In order to characterize the cells in our culture, we have performed experiments in parallel to this study using markers for stemness (Oct4) and endothelial origin (CD34) [40]. Furthermore, we have done clone formation experiments and also applied the commonly used tenocyte markers scleraxis and tenomodulin, which the vast majority of cells were stained positive for [40]. The results therefore suggest that the vast majority of cultured tendon derived cells are differentiated fibroblastic-like cells. However, we cannot completely rule out that the results of the study are to some extent mediated via other cell types that may be present in a very small amount.

Another limitation of this study is that the tissue and cells investigated were taken from pathologic plantaris tendons. A comparison with specimens from healthy individuals would have been desired and helpful for further interpretation. However, the thickness of normal plantaris tendons is extremely small [30]. Taking biopsies from humans without harvesting the whole tendon is considered to be unrealistic. Thus, material can only be taken from patients with indication for plantaris tendon removal, and those often have tendinopathy [30]. To harvest the whole tendon from healthy individuals without symptoms would be unethical. Furthermore, several studies have already reported elevated expression patterns of VGluT2 and NMDAR1 in tendinopathic patellar and Achilles tendons [10–12]. In this study, the purpose of histological analysis was to show the existence of a glutamate signaling. The main focus was put on the in vitro effects of glutamate on tendon cells and the impact of strain and Dex. Future studies should test these findings on other tendons and also compare cells from healthy and pathologic tendons, where this is possible, such as the Achilles and patellar tendons.

## Conclusion

Altogether, it can be concluded that there is some evidence suggesting that glutamate is involved in the processes of tendinopathic tissue features: (1) We have found a non-neuronal glutamate system in the plantaris tendon affected by tendinopathy. (2) Exposure of plantaris tendon derived cells in culture to glutamate/NMDA leads to a decrease of scleraxis, that may, to some extent, indicate a reduction of tenocyte phenotype. (3) Persistent loading, which often is a factor for developing tendinopathy, increases the internal glutamate production and may also increase NMDAR1 receptor expression. (4) Dex may lead to a reduction of glutamate production.

## Abbreviations

CS: Christoph Spang
Dex: Dexamethasone
DMEM: Dulbecco’s Modified Eagle Medium
GLS: Glutaminase
GOT1: Glutamic-Oxaloacetic Transaminase 1
HBSS: Hank’s Balanced Salt Solution
JC: Jialin Chen
LJB: Ludvig Backman
LDH: Lactarte Dehydrogenase
NMDAR1: N-Methyl-D-Aspartate receptor 1
PD: Patrik Danielson
SCX: Scleraxis
SLR: Sandrine Le Roux
VGluT2: Vesicular Glutamate Transport 2
